# Predictors of mortality among children under five with severe acute malnutrition in intensive therapeutic nutrition units in Kasaï Central Province, 2024: a retrospective cohort study

**DOI:** 10.11604/pamj.2026.54.19.50029

**Published:** 2026-05-25

**Authors:** Liévin Tshimanga Kabongo, Bernard-Kennedy Nkongolo Mukendi, Fernand Luenga Mayaya, Valery Biduaya Kuyaku, Espérant Ntambue Malu, Paulin Nkolamoyo Musungula, Paulin Mutombo Beya Wa Bitadi

**Affiliations:** 1 Kasaï Central Provincial Health Division, Kasaï Central, Democratic Republic of Congo,; 2 Institut Supérieur des Techniques Médicales de Kananga, Kananga, République Démocratique du Congo,; 3 School of Public Health, University of Kinshasa, Kinshasa, Democratic Republic of Congo

**Keywords:** Predictors, mortality, child, severe acute malnutrition, intensive therapeutic nutrition units, Kasaï Central

## Abstract

**Introduction:**

severe acute malnutrition affects approximately 19 million children under 5 years of age worldwide. In the Democratic Republic of Congo, between July 2024 and June 2025, approximately 1.4 million children aged 6 to 59 months are estimated to suffer from severe acute malnutrition. It represents a major cause of mortality in low-income countries, particularly in this country. This study aims to identify predictors of mortality among children admitted to the intensive therapeutic nutrition units in Kasaï Central.

**Methods:**

a retrospective cohort study was conducted among 432 children admitted for severe acute malnutrition (SAM) in six intensive therapeutic nutrition units in Kasaï Central, from January to November 2024. Data were collected via KoboCollect, cleaned in Excel, and analyzed with SPSS. Survival analyses (Kaplan-Meier) and a multivariate Cox model were used to identify predictors of mortality.

**Results:**

mortality was 6.3%, and median survival time was 7 days. Weight gain ≥100 g was more common in survivors (51.9%) than in non-survivors (33.3%). Significant predictors included sepsis (HRa = 3.325), hypoglycemia (HRa = 4.324), anemia (HRb = 2.32), and dehydration (HRa = 1.726).

**Conclusion:**

early detection of comorbidities and their prompt management are essential to improve the survival of children with SAM. Specific strategies targeting the main complications must be strengthened in the Intensive Therapeutic Nutrition Units of Kasaï Central.

## Introduction

Severe acute malnutrition (SAM) remains a global public health emergency. In the Democratic Republic of Congo (DRC), the nutritional situation remains critical. The Integrated Phase Classification (IPC) 2024-2025 analysis estimates that more than 4.45 million children aged under 5 months will suffer from acute malnutrition, including 1.39 million cases of SAM requiring urgent care, in addition to 3.7 million pregnant or breastfeeding women affected between July 2024 and June 2025 [1]. According to the Demographic and Health Survey (DHS, 2023-2024) [2], the national prevalence of global acute malnutrition (GAM) among children under five is 7.2%, including 1.9% of SAM. In Kasaï Central, these rates are slightly lower but worrying, with 5.3% GAM and 1% SAM [2].

Intensive therapeutic nutrition units (UNTIs) are hospital facilities specialized in the care of children suffering from SAM with medical complications. They are generally integrated into general referral hospitals or equipped health centers, and operate according to the protocols of the National Nutrition Program [3]. Treatment includes medical stabilization (treatment of infections, rehydration, correction of metabolic imbalances), nutritional rehabilitation based on ready-to-use therapeutic foods, and psychosocial support for families. In the DRC, UNTI attendance varies by province, but remains particularly high in areas with a high prevalence of SAM. In Kasaï Central, UNTIs are often saturated, faced with stockouts, a lack of qualified personnel, and difficult accessibility for displaced or rural populations [1,3].

Kasaï Central Province is a region facing chronic food insecurity, recurrent armed conflicts, population displacements, and frequent epidemics (measles, cholera, monkeypox) [4]. These factors worsen access to care, particularly in rural areas where health facilities are often inadequate. In 2023, more than 1.1 million children and 605,000 pregnant or breastfeeding women required nutritional care [4]. illustrating the scale of the needs and the pressure on the local health system. Several health zones in the province regularly exceed the emergency threshold of 15% SAM prevalence [1].

Although African literature documents this form of malnutrition as well as the risk factors for mortality, this issue remains poorly documented in the UNTIs of Kasaï Central. Yet, such information would help guide regional nutrition policies.

This study, therefore, aimed to identify the clinical, nutritional, and socio-environmental determinants associated with mortality among children admitted for SAM in UNTIs between July 2024 and June 2025, in order to optimize treatment protocols and strengthen operational capacities in a context of prolonged humanitarian crisis.

## Methods

**Study framework:** the study focused on six UNTIs in Kasaï Central, mostly located in rural areas. Care for children suffering from SAM is concentrated at the Saint George's General Referral Hospital (GRH) and the Mikalayi GRH. Some UNTIs are informal, but integrated due to their active role in treatment.

**Population and selection criteria:** the target population consisted of children under five years of age suffering from SAM and admitted to the province's UNTIs between January and November 2024. The inclusion criteria included children who presented with SAM, as diagnosed according to the standards of the national nutrition program, based on available data. Medical records were excluded if they were incomplete, i.e., if they did not include essential information for analysis (age, sex, date of admission, hospitalization outcome, clinical and nutritional parameters), or if patients had left the hospital against medical advice.

**Sampling:** the study focused on a total of 432 medical records of children treated for SAM between January and December 2024. The selection process for the survey sites was structured around four successive stages. First, the province of Kasaï Central was chosen as the study setting, in accordance with the study objectives and due to the high prevalence of SAM observed in this region. Then, six health zones were targeted among those benefiting from the support of the multisectoral nutrition and health program, which ensured favorable technical and logistical support for data collection. In each of these zones, a UNTI was selected, taking into account its reception capacity and the availability of medical records. Finally, all the records of children admitted for SAM in these six structures during the defined period were included exhaustively, guaranteeing complete coverage of cases hospitalized in this setting.

**Study variables:** the dependent variable was the outcome of hospitalization, defined as the survival or death of the child. The independent variables included sociodemographic characteristics (age, sex, area of residence, and vaccination status), clinical signs observed on examination (fever, lethargy, edema, diarrhea, signs of shock, and conjunctival pallor), paraclinical parameters (glycemia, hemoglobin level), therapeutic modalities administered (antibiotics, F75/F100 therapeutic diet, and intravenous infusion), as well as nutritional indicators (weight gain and appetite).

**Data collection:** the data were extracted from the paper medical records of children followed in the UNTIs, using a standardized collection form designed and deployed on KoboCollect to systematically record all information required for the study.

**Processing and analysis:** data were cleaned in Excel, coded, and analyzed using SPSS version 29.0 software. Descriptive statistics were used to characterize the study population according to sociodemographic, clinical, and nutritional variables. Frequencies, means, and standard deviations were calculated for quantitative variables, while proportions were used for qualitative variables.

The survival time of children hospitalized for severe acute malnutrition was estimated using Kaplan-Meier curves, with median survival calculated. Comparisons between groups were performed using the log-rank test.

Finally, a multivariate Cox regression model was used to identify predictors independently associated with mortality, adjusting for relevant covariates (age, sex, type of malnutrition, presence of complications, maternal education level, etc.). The threshold for statistical significance was set at p < 0.05.

**Ethical aspect:** all ethical considerations were respected in accordance with the Declaration of Helsinki. The study was approved by the Ethics Committee of the School of Public Health of Kinshasa (approval reference: ESP/CE/144/2024). Favorable opinions were also obtained from the heads of the concerned UNTIs.

## Results

**Sociodemographic characteristics:** among the 432 children with severe acute malnutrition, the majority were admitted to Saint Georges (31.9%) and Mikalayi (24.1%). Approximately 60% of the UNTIs were located in rural areas. The gender distribution shows a slight male predominance (53.9%), and 62.5% of the children were between 24 and 59 months, indicating a marked prevalence in this age group (Table 1).

**Table 1:** distribution of children aged 0 to 59 months with severe acute malnutrition according to their sociodemographic characteristics, Kasaï-Central province, December 2024

Sociodemographic characteristics	Frequency (n=432)	Percentage (%)
**Names of UNTI**		
Bunkonde GRH	66	15.3
Demba GRH	68	15.7
Lukonga GRH	35	8.1
Mikalayi GRH	104	24.1
SAINT GEORGES GRH	138	31.9
TSHIKAJI GRH	21	4.9
**Location of UNTI**		
Rural	259	60.0
Urban	173	40.0
**Sex**		
Female	199	46.1
Male	233	53.9
**Age (in month)**		
6-11	67	15.5
12-23	95	22.0
24-59	270	62.5


UNTI: intensive therapeutic nutrition unit; GRH: General Referral Hospital

**Figure 1:**
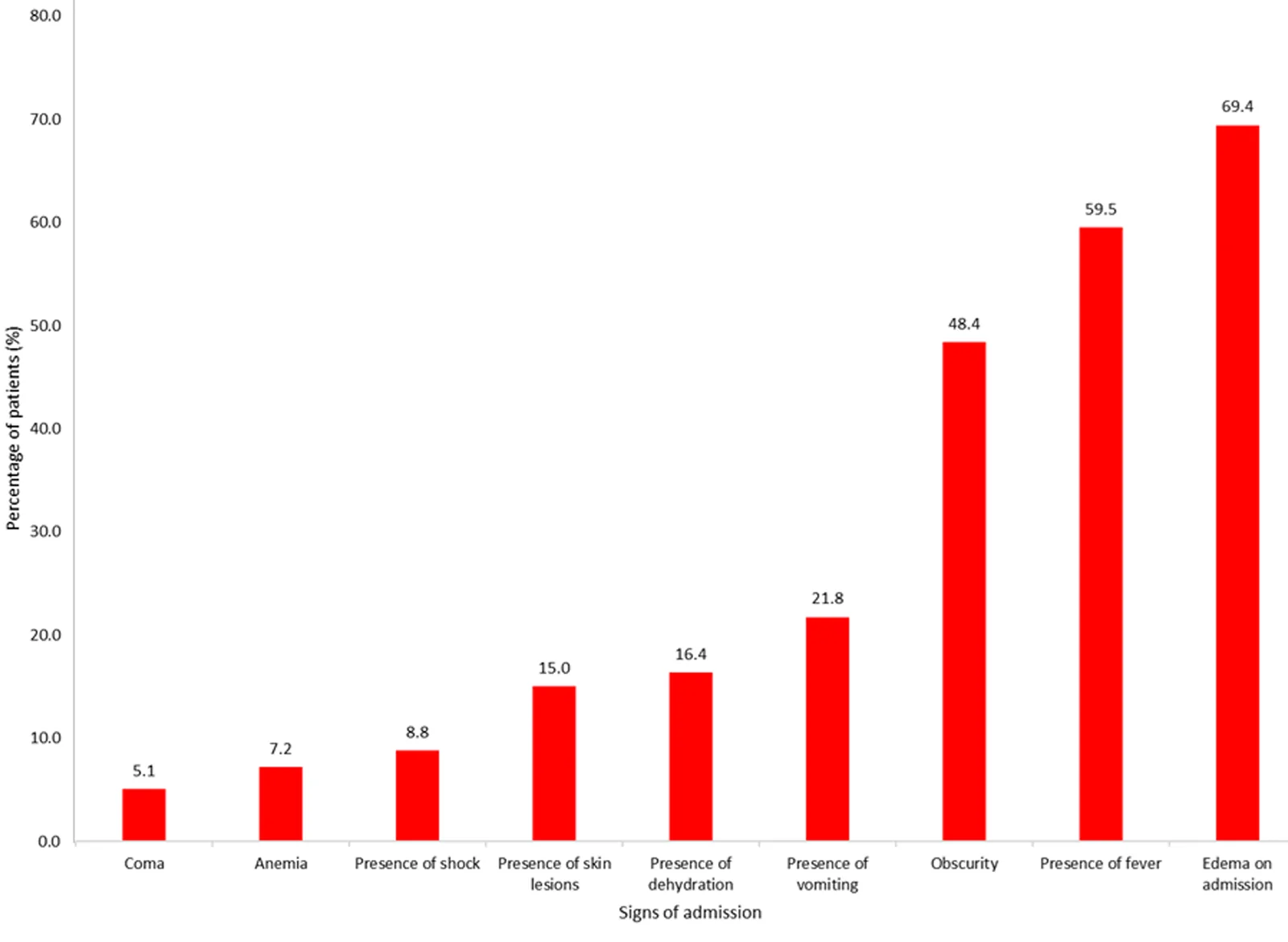
distribution of severe acute malnutrition cases in children under five years of age according to their clinical profile upon admission to the UNTIs in the province of Kasaï Central, December 2024

**Clinical profile at admission and outcome of SAM children:** at hospitalization, 69.4% of children presented with edema and 59.5% with fever. Other signs of severity were common: obtundation (48.4%), vomiting (21.8%), dehydration (16.4%), skin lesions (15.0%), shock (8.8%), anemia (7.2%), and coma (5.1%); the median duration of hospitalization was 7 days, and the median weight gain was 100 grams. (Figure 1). Of 100 SAM children admitted to the UNTI, 6,2% had died.

**Bivariate analysis of predictors associated with mortality:** bivariate analysis (Table 2) identified UNTI site (p = 0.003), rural location (p = 0.002), and age (p = 0.043) are significantly associated with mortality. Mortality rates were higher at the Mikalayi site (n=12). Children aged 24–59 months (n=22) and a new admission (n=22) experienced the highest mortality rate (p< 0.05). Gender and other complications were not statistically significant.

**Table 2:** clinical and sociodemographic factors associated with mortality according to the Chi-square test in the UNTIs of the province of Central Kasaï, December 2024

Variables	Deceased (n=27)	Cured (n=405)	Total (n=432)	p-value
**Name of UNTI**				**0.003**
Bunkonde GRH	6 (9.1%)	60 (90.9%)	66 (100%)	
Demba GRH	4 (5.9%)	64 (94.1%)	68 (100%)	
Lukonga GRH	2 (5.7%)	33 (94.3%)	35 (100%)	
Mikalayi GRH	12 (11.5%)	92 (88.5%)	104 (100%)	
Saint Georges GRH	1 (0.7%)	137 (99.3%)	138 (100%)	
Tshikaji GRH	2 (9.5%)	19 (90.5%)	21 (100%)	
**Location of UNTI**				**0.002**
Rural	24 (9.3%)	235 (90.7%)	259 (100%)	
Urban	3 (1.7%)	170 (98.3%)	173 (100%)	
**Gender of the child**				**0.57**
Female	15 (7.5%)	184 (92.5%)	199 (100%)	
Male	12 (5.2%)	221 (94.8%)	233 (100%)	
**Age (in month)**				**0.043**
6-11	0 (0%)	67 (100%)	67 (100%)	
12-23	5 (5.3%)	90 (94.7%)	95 (100%)	
24-59	22 (8.1%)	248 (91.9%)	270 (100%)	
**Type of admission**				**0.005**
New admission	22 (5.3%)	392 (94.7%)	414 (100%)	
Readmission	1 (20%)	4 (80%)	5 (100%)	
Relapse	4 (30.8%)	9 (69.2%)	13 (100%)	
**Medical complications**				**0.25**
Yes	20 (7.2%)	256 (92.8%)	276 (100%)	
No	7 (4.5%)	149 (95.5%)	156 (100%)	
**Weight gain (g)**				**1.15**
Weight loss (<0)	17 (8.6%)	180 (91.4%)	197 (100%)	
Between 0 and 100 g	1 (6.3%)	15 (93.7%)	16 (100%)	
≥ 100 g	9 (4.1%)	210 (95.9%)	219 (100%)	
**Length of hospitalization**				**0.46**
< 7 days	18 (6.9%)	241 (93.1%)	259 (100%)	
≥ 7 days	9 (5.2%)	164 (94.8%)	173 (100%)	

UNTI: intensive therapeutic nutrition unit; GRH: General Referral Hospital

**Complications associated with mortality:** clinical complications such as malaria (p=0.027), edema, anemia, and dehydration (p=0.001) were the main causes of death. Sepsis, hypoglycemia, edema, dehydration, anemia, and malaria were all significantly associated with the clinical outcome of hospitalized children (Table 3).

**Table 3:** distribution of severe acute malnutrition children aged 0 to 59 months according to Complications based on hospitalization outcome in UNTIs in the province of Central Kasaï, December 2024

Complications	Issue in hospitalization			p-value
	Death (n = 27)	Healing (n = 405)	Total (n = 432)	
**Pneumonia**				**0.11**
Yes	6 (22.2%)	51 (12.6%)	57 (13.2%)	
No	21 (77.8%)	354 (87.6%)	375 (86.8%)	
**Sepsis**				**0.001**
Yes	11 (40.7%)	16 (4.0%)	27 (6.3%)	
No	16 (59.3%)	389 (96.0%)	405 (93.8%)	
**Hypoglycemia**				**0.001**
Yes	8 (29.6%)	13 (3.2%)	21 (4.9%)	
No	19 (70.4%)	392 (96.8%)	411 (95.1%)	
**Digestive candidiasis**				**0.22**
Yes	5 (18.5%)	50 (12.4%)	55 (12.7%)	
No	22 (81.5%)	355 (87.6%)	377 (87.3%)	
**Cough**				**0.56**
Yes	2 (7.4%)	38 (9.4%)	40 (9.3%)	
No	25 (92.6%)	367 (90.6%)	392 (90.7%)	
**Anemia**				**0.001**
Yes	15 (55.6%)	45 (11.1%)	60 (13.9%)	
No	12 (44.4%)	360 (88.9%)	372 (86.1%)	
**Meningitis**				**0.58**
Yes	1 (3.7%)	13 (3.2%)	14 (3.2%)	
No	26 (96.3%)	392 (96.8%)	418 (96.8%)	
**Edema at discharge**				**0.001**
Yes	15 (55.6%)	93 (23.0%)	108 (25.0%)	
No	12 (44.4%)	311 (77.0%)	323 (75.0%)	
**Diarrhea**				**0.49**
Yes	4 (14.8%)	53 (13.1%)	57 (13.2%)	
No	23 (85.2%)	351 (86.9%)	374 (86.6%)	
**Dehydration**				**0.001**
Yes	12 (44.4%)	62 (15.3%)	74 (17.1%)	
No	15 (55.6%)	343 (84.7%)	358 (82.9%)	
**Malaria**				**0.027**
Yes	22 (81.5%)	249 (61.6%)	271 (62.7%)	
No	5 (18.5%)	156 (38.6%)	161 (37.3%)	

**Figure 2:**
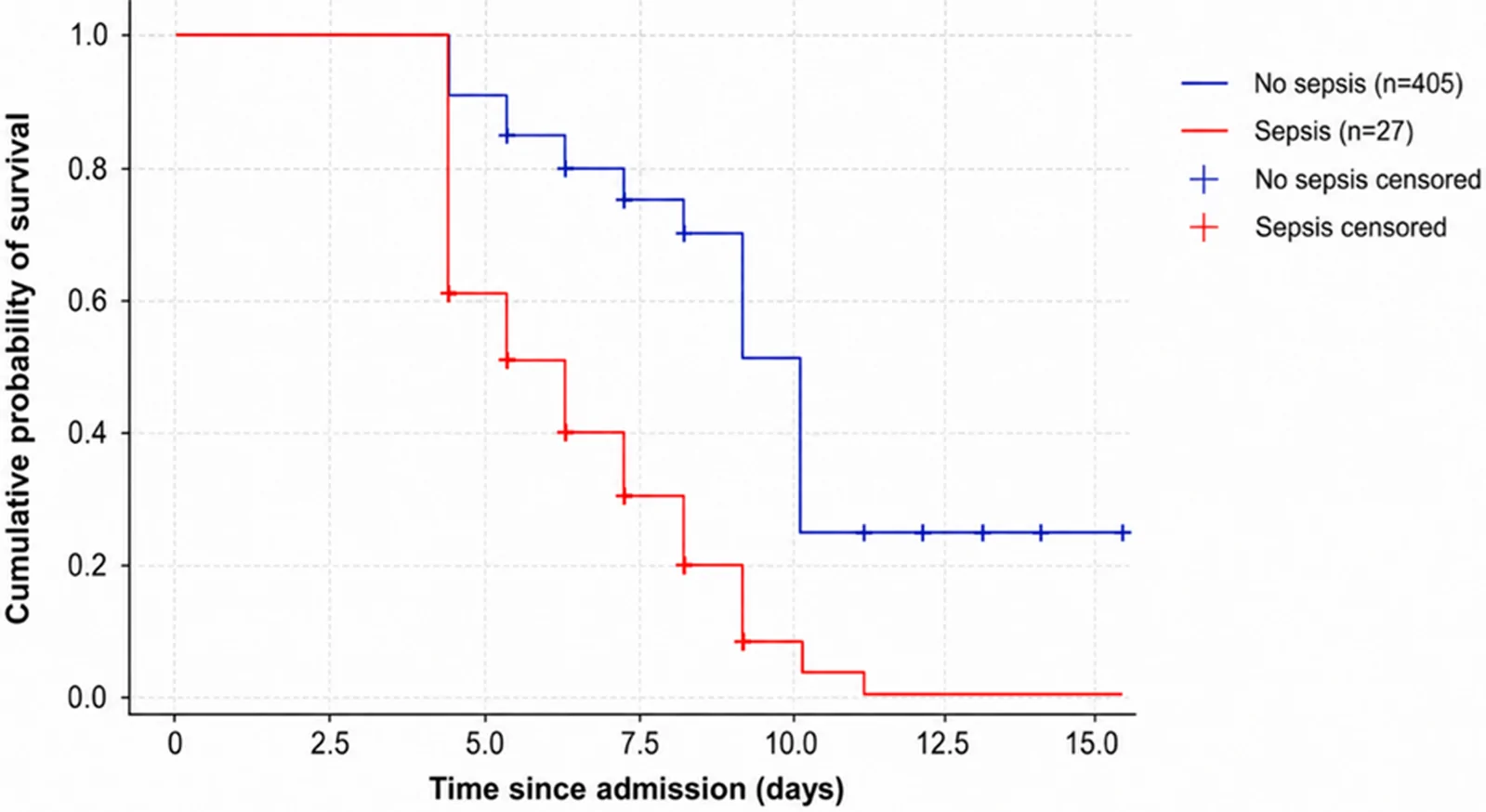
Kaplan-Meier survival curve of children aged 0 to 59 months in UNTIs in the sepsis group and the non-sepsis group, Kasaï-Central province, December 2024

**Survival Analysis:** the Kaplan-Meier curve (Figure 2) illustrates a rapid decline in survival among children with sepsis. Survival is significantly better among those without sepsis, highlighting the critical impact of this infection.

**Multivariate analysis of predictors of mortality:** multivariate analysis by Cox model (Table 4) confirms that sepsis (adjusted Hazard Ratio (aHR) = 3.325, p=0.009), hypoglycemia (aHR = 4.324, p=0.002), anemia (aHR = 2.896, p=0.035), and dehydration (aHR = 1.726, p=0.024) are independent predictors of mortality. These results reinforce the importance of rapid diagnosis and targeted management of complications upon admission to the NICU.

**Table 4:** Cox regression in bivariate and multivariate analysis of predictors of mortality in children aged 0 to 59 months admitted to UNTIs in Kasaï Central, December 2024

Variables of the equation	HRb	95% CI		p	HRa	95% CI		
		Lower	Superior			Lower	Superior	P
**Gender of the child**								
Male	0.9	0.8	1.1	0.502	0.585	0.261	1,314	0.194
Female	1				1			
**Age group (in month)**								
6-11	1				0.004	0.000	3,430	0.574
12-23	1.1	0.9	1.5	0.373	0.546	0.222	1,345	0.188
24-59	1	0.8	1.2	0.822	1,000			
**Pneumonia**								
Yes	1.1	0.8	1.4	0.703	1,455	1,268	2,771	0.003
No	1				1,000			
**Sepsis**								
Yes	1.5	1.03	2.8	0.003*	3,325	1,245	8,880	0.009
No	1				1,000			
**Hypoglycemia**								
Yes	1.6	1.4	2.1	0.037 *	4,324	1,680	11,132	0.002*
No	1				1,000			
**Dehydration**								
Yes	2.7	1.5	3.9	0.011*	1,726	1,549	3,958	0.024
No	1				1			
**Anemia**								
Yes	7,364	3,579	15,155	0.001*	2,896	1,078	7,779	0.035*
No	1				1,000			

* : P value less than 0.005

## Discussion

**Key results:** the objective of this study was to identify factors associated with mortality among children admitted for severe acute malnutrition to the UNTIs of Kasaï Central. The analysis of 432 medical records of children admitted for severe acute malnutrition to the UNTIs of Kasaï Central revealed three main lines of evidence. First, the incidence of infant mortality observed in these facilities was 6%, with deaths concentrated among children aged 24 to 59 months, occurring mainly during the first week of hospitalization. Second, multivariate analysis identified several determinants significantly associated with mortality, including female sex, rural residence, lack of measles vaccination, lethargy, conjunctival pallor, hypoglycemia, and anemia, while diarrhea and dehydration did not show a significant association after adjustment.

**Incidence and characteristics of infant mortality in the UNTI of Central Kasaï:** the infant mortality observed in the UNTIs of Kasaï Central in 2024 was 6.3%, reflecting the extreme vulnerability of children admitted for SAM. This mortality rate was particularly concentrated among children aged 24 to 59 months, with 81.5% of deaths occurring during the first week of hospitalization, suggesting a critical failure in initial case management. Excess mortality among girls has also been observed. Although females generally have a biological survival advantage in early childhood, this advantage may be attenuated in the context of severe acute malnutrition [5,6]. Evidence from low- and middle-income countries suggests that girls are often brought later to health services and present with more advanced disease at admission, reflecting gender-based disparities in healthcare-seeking behavior and access [7]. Such delays in care-seeking may contribute to higher mortality among girls in nutritional rehabilitation programs [8].

The mortality rate reported in this study (6.3%) is lower than that reported in the South Kivu Province (9.2%) [9] and substantially lower than that of a study conducted in Kabinda, where hospital case fatality for SAM reached 24.2% [10]. This contrast highlights significant variations in prognosis according to local contexts and care structures. Short-term outcomes of SAM largely depend on the type of malnutrition. In uncomplicated SAM, hospital mortality is generally below 5%, whereas it may range from 10% to 40% in complicated SAM [11].

At the international level, hospital mortality associated with SAM also shows considerable variability: 11.3% (95% CI: 8.8-13.7) in Ethiopia and 25.2% (95% CI: 19.9-30.4) in Kampala, Uganda [12,13]. These differences reflect variations in quality of care, access to health services, and timeliness of management, all of which are critical determinants of prognosis for malnourished children.

These findings indicate limited effectiveness of interventions during the intensive phase of SAM management and highlight the urgent need to improve vaccination coverage, early screening, and rapid referral to appropriate facilities [9]. An integrated approach, combining curative care, prevention, and community mobilization, therefore appears essential for reducing infant mortality associated with SAM.

**Predictors of mortality in the UNTI of Kasaï Central:** the study results highlight the critical role of certain metabolic and infectious complications in the mortality of children with SAM. The primary conditions associated with fatal outcomes include sepsis, hypoglycemia, anemia, and, to a lesser extent, dehydration. These pathologies reflect extreme physiological vulnerability, linked to the immune and energy deficits characteristic of SAM. Sepsis rapidly compromises vital functions in immunocompromised children due to the systemic inflammatory response and potential multiorgan failure it can induce [14].

These findings are consistent with those of Mwanza *et al*. and Chisti *et al*. [10,15], who also demonstrated a significant association between sepsis and mortality in SAM. Similarly, Ledger *et al*. and Mwanza *et al*. [10,16] reported that hypoglycemia is a major predictor of death in severely malnourished children. Regarding anemia, observations by Moges *et al*. and Venigalla *et al*. [17,18] confirm its exacerbating role on survival outcomes. These convergences reinforce the notion that the combination of severe malnutrition, serious infections (notably sepsis and pneumonia), hypoglycemia, and anemia exponentially increases infant mortality in low- and middle-income countries. Regarding dehydration, although it is frequently cited as a risk factor for death in malnourished children [19], its association with mortality was not confirmed after adjustment in this study. This may reflect improvements in rehydration management within the UNTIs studied.

Finally, these complications are often detected late due to delayed consultation, insufficient recognition of severity signs, or gaps in clinical skills [9]. Such delays compromise the timeliness and quality of care, leading to preventable deaths. These findings underscore the need for systematic and early screening of these complications upon admission to UNTIs. Implementing standardized protocols for initial assessment and continuous clinical monitoring can enable the rapid identification of at-risk children, thereby limiting preventable deaths. Training healthcare staff to recognize signs of severity is a key element in improving responsiveness and quality of care.

**Study limitations:** this study had certain limitations that may have influenced the reported results. First, the data were extracted from the medical records of children admitted to the studied UNTIs. In the DRC, these sources have significant limitations due to the limited storage capacity and organization of medical archives. A careful review of these records, along with regular checks conducted by multisectoral nutrition and health program and national nutrition program staff within the framework of service purchases, helped ensure their reliability. Second, the lack of post-hospitalization follow-up prevents the assessment of medium-term outcomes, such as relapses or deaths occurring after discharge, thereby introducing an attrition bias that limits the scope of conclusions regarding the overall effectiveness of care. Nevertheless, this study provided evidence-based data in the local context, thereby facilitating decision-making. The exhaustive data collection in functional UNTIs, where records are maintained regularly according to the national nutrition program protocols, represents a major strength. Furthermore, the substantial sample size helped to reinforce the internal validity of the results.

## Conclusion

This study highlights the central role of sepsis, hypoglycemia, anemia, and severe acute malnutrition as major predictors of infant mortality in UNTIs in Kasaï Central. It underscores the urgent need for early medical care, enhanced screening, and appropriate nutritional rehabilitation. Improved care protocols and expanded access to health services are essential to reduce preventable deaths. Finally, it calls for structural interventions targeting socioeconomic inequalities and community vulnerabilities

### What is known about this topic

- Severe acute malnutrition is a major cause of child morbidity and mortality in sub-Saharan Africa;
- Intensive therapeutic nutrition units play a central role in the care of severely malnourished children;
- Severe infections such as sepsis, dehydration, and hypoglycemia are frequently associated with fatal outcomes in this vulnerable population.

### What this study adds

- The study identifies independent predictive factors of mortality among children with severe acute malnutrition (SAM) admitted to intensive nutritional therapy units (UNTIs) in Kasaï Central;
- The study highlights the critical role of certain clinical comorbidities, particularly sepsis, hypoglycemia, anemia, and dehydration, in the occurrence of death;
- The findings underscore the need to strengthen clinical monitoring and ensure the early management of these complications to improve the survival of children hospitalized for SAM; finally, it provides recent local data that can inform nutrition policies and programs in the province and, more broadly, across the Democratic Republic of Congo.
